# Galectin-3 and Strain Imaging for Early Heart Failure Prediction After First Myocardial Infarction

**DOI:** 10.3390/ijms262110718

**Published:** 2025-11-04

**Authors:** Małgorzata Sikora-Frąc, Grażyna Sygitowicz, Ewa Pilichowska-Paszkiet, Krzysztof Smarż, Paweł Maciejewski, Piotr Kokowicz, Marta Prządka, Andrzej Budaj, Beata Zaborska

**Affiliations:** 1 Department of Cardiology, Centre of Postgraduate Medical Education, Grochowski Hospital, 51/59 Grenadierów Str., 04-073 Warsaw, Poland; msikora@cmkp.edu.pl (M.S.-F.); epilichowska@cmkp.edu.pl (E.P.-P.); ksmarz@cmkp.edu.pl (K.S.); pmaciejewski@cmkp.edu.pl (P.M.); pkokowicz@cmkp.edu.pl (P.K.); marta.przadka@gmail.com (M.P.); abudaj@cmkp.edu.pl (A.B.); bzaborska@cmkp.edu.pl (B.Z.); 2 Department of Laboratory Medicine, Faculty of Pharmacy, Medical University of Warsaw, 1 Banacha Str., 02-097 Warsaw, Poland

**Keywords:** galectin-3, heart failure, acute myocardial infarction, left ventricular global longitudinal strain, left atrial reservoir strain

## Abstract

Galectin-3 (Gal-3), a biomarker of fibrosis, is involved in post-infarction remodelling, but its short-term prognostic value remains uncertain. This study aimed to evaluate the prognostic value of Gal-3 for new-onset heart failure (HF) in first acute myocardial infarction (MI) during the in-hospital phase following MI and to assess its association with advanced echocardiographic indices of myocardial and atrial dysfunction, including left ventricular global longitudinal strain (LVGLS) and left atrial reservoir strain. In this prospective study, 105 consecutive patients with STEMI/NSTEMI (mean age 61 ± 11 years) were enrolled. New-onset HF, defined by symptoms, elevated NT-proBNP, and echocardiographic LV dysfunction, developed in 34 patients (32%) during follow-up of a median of 10 [8–13] days. Median serum Gal-3 concentration was 11.6 [9.5–13.5] ng/mL. Gal-3 correlated with echocardiographic indices of myocardial and atrial dysfunction (*p* = 0.001). Receiver operating characteristic analysis showed moderate discriminative ability (AUC = 0.712; cut-off > 10.9 ng/mL). In multivariable regression, both Gal-3 and LVGLS independently predicted HF, and their combination improved discrimination (AUC = 0.833). In conclusion, Gal-3, particularly when combined with LVGLS, is a valuable early prognostic marker of new-onset HF during the in-hospital phase of acute MI. The combined assessment of Gal-3 and GLS provides a novel, translational biomarker–imaging approach to post-MI prognosis.

## 1. Introduction

Ischaemic heart disease and myocardial infarction (MI) remain leading causes of heart failure (HF) due to myocardial injury [1]. The development of HF during the acute, in-hospital phase of MI is associated with a markedly worse prognosis and necessitates early therapeutic modification. Therefore, accurate early risk stratification is essential. Extensive research has been directed toward integrating biochemical and imaging biomarkers to improve prognostic assessment in this setting. Galectin-3 (Gal-3), a soluble β-galactoside-binding lectin widely expressed in human tissues, has emerged as a key mediator of myocardial remodelling and fibrosis [2,3,4,5]. Unlike “bystander” biomarkers like N-terminal pro B-type natriuretic peptide (NT-proBNP) or C-reactive protein, Gal-3 plays a causal role (“culprit” biomarker) by promoting fibroblast proliferation and collagen deposition [5,6]. Elevated Gal-3 concentration has been shown to predict mortality and HF-related hospitalisation, independent of traditional risk factors [7,8,9]. Although Gal-3 is well established as a prognostic marker in chronic HF, evidence in the setting of acute coronary syndrome remains limited [10,11]. Experimental data suggest a biphasic role: in early MI Gal-3 may cause cytoprotective and anti-inflammatory effects, whereas persistent elevation in the later phase contributes to chronic inflammation, fibrosis, and adverse ventricular remodelling [12,13]. Clinical studies also indicate that elevated Gal-3 concentration is associated with unfavourable in-hospital outcomes in ST-segment elevation MI (STEMI) and, moreover, is related to left ventricular (LV) remodelling, HF hospitalisation, and cardiovascular death in mid-term and long-term follow-up [14,15,16,17,18,19,20,21]. However, prospective data on the prognostic value of Gal-3 for new-onset HF in acute MI are lacking. Transthoracic echocardiography (TTE) remains fundamental in patients with acute coronary symptoms and patients with suspected HF [1,22].

Standard TTE assessment of systolic function, in particular, left ventricular ejection fraction (LVEF), and diastolic function proposes powerful parameters for risk stratification after acute MI. Advanced TTE modalities, especially speckle tracking echocardiography (STE)-derived longitudinal strain, can provide additional prognostic value beyond standard techniques [23].

Combining imaging and biochemical biomarkers, such as Gal-3, with advanced echocardiographic parameters could improve diagnostic and prognostic accuracy [24].

Therefore, in the present study, we aimed to examine the prognostic value of Gal-3 for new-onset HF in acute MI during the in-hospital phase. To explore the potential use of combined biochemical and imaging biomarkers, the association between Gal-3 concentrations and advanced echocardiographic indices of myocardial dysfunction was assessed.

## 2. Results

### 2.1. Patient Characteristics

We screened 661 pts, of whom 105 (81 males (77%), mean age 61 ± 11 years) met the inclusion criteria and were enrolled in the study. The remaining 556 subjects were excluded due to not meeting entry criteria. During hospitalisation, new-onset HF was diagnosed in 34 out of 105 patients (32%). Two of the thirty-four patients developed symptoms of acute HF, and one of them required a catecholamine infusion. Baseline characteristics, comorbidities, treatment details, and laboratory parameters for the entire study population, as well as comparisons between patients with and without HF, are presented in Table 1. Patients who developed HF were significantly older and exhibited poorer renal function compared to those without HF. STEMI accounted for 56.1% of all MI in the overall cohort, with a significantly higher prevalence in the HF group (70.6%) compared to the non-HF group (49.3%); *p* = 0.04. The culprit lesion was identified in the left anterior descending artery (LAD) in 50.5% of all patients, with a higher prevalence in the HF group (70.6%) compared to the non-HF group (40.8%; *p* = 0.004). Revascularisation was successful and complete in all patients. Patients with HF had longer hospital stay and were more frequently treated with diuretics, sodium–glucose co-transporter 2 (SGLT-2) inhibitors, and aldosterone antagonists and were less frequently treated with prasugrel in comparison to those without HF.

The echocardiographic parameters for the entire cohort, as well as subgroup comparisons between HF and non-HF patients, are summarised in Table 2. Patients with HF had significantly lower LVEF, absolute LV global longitudinal strain (GLS) value, left atrial reservoir strain (LARS), and tricuspid annular plane systolic excursion (TAPSE) mean early diastolic tissue velocities (e′) and higher E/e′ ratio.

### 2.2. Gal-3 Concentration and Other Biomarkers

For all study subjects, the median plasma Gal-3 concentration was 11.6 ng/mL [9.5–13.5]. Gal-3 concentration was significantly higher in patients who presented new-onset HF compared to those without HF (*p* < 0.001). Peak Troponin T and NT-proBNP concentrations were also significantly elevated in the HF group (both *p* < 0.001; Table 1). A statistically positive significant correlation was observed between Gal-3 concentration and NT-proBNP (r = 0.39; *p* < 0.001), as well as age (r = 0.33; *p* = 0.001). No statistically significant correlation was observed between Troponin T concentration and Gal-3 concentration (r = 0.18, *p* = 0.076).

### 2.3. Echocardiographic Parameters in Relation to Biomarkers

**Figure 1:**
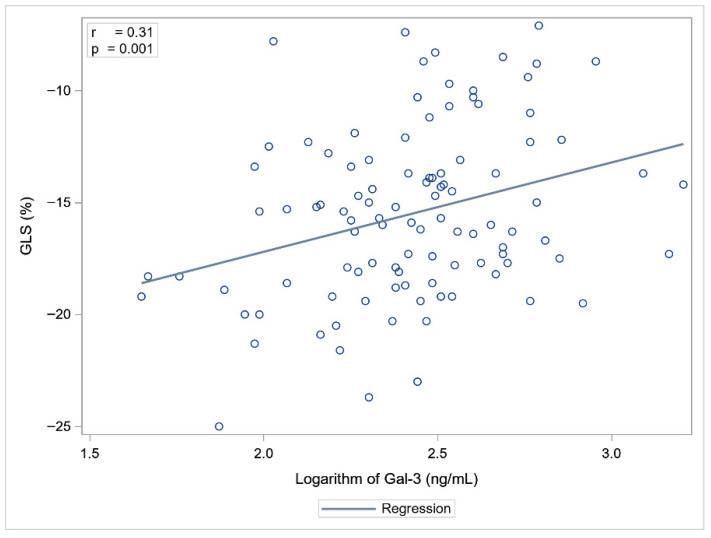
Correlation between Gal-3 and GLS.

**Figure 2:**
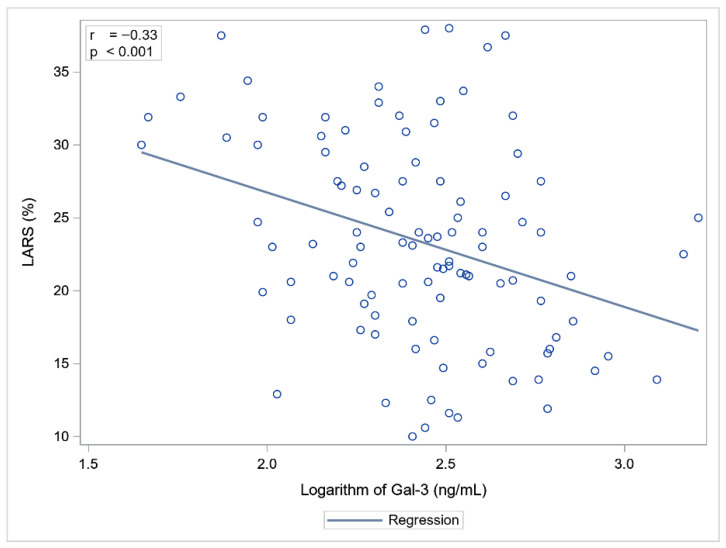
Correlation between Gal-3 and LARS.

Statistically significant correlations were observed between Gal-3 and NT-proBNP concentrations and LVEF, GLS, and LARS. NT-proBNP showed a slightly stronger association with cardiac functional parameters compared to Gal-3 (Table 3, Figure 1 and Figure 2).

Stratification of patients into tertiles, based on LV and LA function, revealed significant differences in Gal-3 concentrations across all tertiles (Table 4). Higher Gal-3 concentrations were associated with poorer LV and LA functional parameters.

Linear regression analysis revealed that Gal-3 and Troponin T, but not NT-proBNP, were independent predictors of echocardiographic parameters of myocardial function (Table 5). Higher Troponin T and Gal-3 concentrations were significantly associated with impaired LV systolic function, as reflected by increased (less negative) GLS values. In terms of LA function, Troponin T and Gal-3 concentrations were significantly negatively associated with LARS values (Table 5).

### 2.4. Gal-3 Prognostic Value in STEMI/NSTEMI

**Figure 3:**
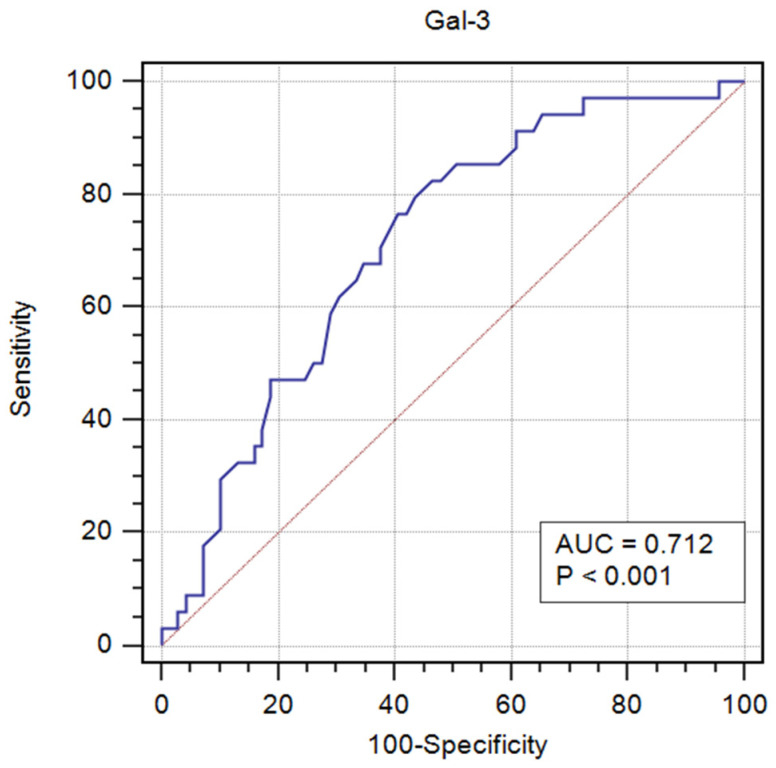
ROC curve analysis of Gal-3 serum concentration in the diagnosis of new-onset HF in acute MI.

Receiver operator characteristic (ROC) curve analysis revealed diagnostic significance of Gal-3 for new-onset HF in acute MI. The area under the curve (AUC) was 0.712 (95% CI: 0.609–0.814; *p* < 0.001), indicating a moderate discriminatory ability of this parameter (Figure 3). Based on the analysis of the maximum Youden index, the optimal cut-off value for Gal-3 was determined to be above 10.9 ng/mL, at which a sensitivity of 82.4% and a specificity of 53.6% were achieved.

Univariate binary logistic regression analysis identified elevated serum creatinine, increased Gal-3 concentration, and reduced GLS as the strongest risk factors for the development of HF in acute MI (Table 6). A 1-unit increase in Gal-3 concentration was significantly associated with a 20% increase of the risk of HF. Similarly, an increase in GLS value was significantly associated with a higher risk of developing HF.

In multivariable binary logistic regression analyses with stepwise selection, both GLS and Gal-3 were independently related to the development of HF (Table 7). The combined model, incorporating both parameters, demonstrated a good discriminative ability, with an AUC of 0.833.

## 3. Discussion

The main finding of our study was that Gal-3 demonstrated a good prognostic value in predicting in-hospital new-onset HF in patients with a first STEMI or NSTEMI successfully treated with primary percutaneous coronary intervention (pPCI). The combined diagnostic model, incorporating GLS as an imaging parameter reflecting LV systolic dysfunction together with Gal-3, revealed a better discriminative ability, with an AUC of 0.833.

Gal-3 is expressed through activation of monocytes/macrophages and participates in the regulation of inflammatory processes and profibrotic pathways, acting as a mediator of myocardial fibrosis in the presence of cellular necrosis and/or apoptosis and the resulting inflammatory reactions. These mechanisms are referred to as the reparative fibrosis pathway [25]. Another mechanism related to collagen synthesis is also known: the reactive pathway, in which fibrosis is secondary to neurohumoral activation without cardiomyocyte necrosis, involving the following cellular mediators: angiotensin II, aldosterone, and endothelin-1 [26]. Gal-3, binding to various ligands, is involved in the regulation of numerous pathophysiological phenomena, not only inflammation and fibrosis but also angiogenesis and apoptosis. These processes are crucial in myocardial remodelling [27,28]. As a regulatory protein, it is elevated in both acute and chronic HF and is involved in the post-injury inflammatory pathway leading to myocardial tissue remodelling [5].

The study revealed that elevated Gal-3 concentrations were observed in patients who developed new-onset HF during hospitalisation after acute STEMI/NSTEMI. There is limited evidence regarding such associations in the early phase after MI. In a retrospective subanalysis of the CLARITY-TIMI 28 trial involving patients with STEMI, Gal-3 was associated with an increased risk of developing HF within 30 days post-myocardial infarction (OR 1.5 (95% CI: 1.24–1.82); *p* < 0.001) [29]. Other studies have investigated the relationship between Gal-3 concentration and the onset of HF in MI patients over a longer follow-up time. Dekleva et al. found higher serum Gal-3 concentration in patients with HF compared to patients without HF six months after MI (*p* = 0.024) [30]. Lorenzo-Almorós et al. revealed that, in patients with STEMI or NSTEMI, Gal-3 concentrations above 6.49 ng/mL were associated with an increased risk of MACE defined as acute ischaemic events, HF, or death (HR 3.26 (95% CI: 1.32–8.04); *p* = 0.010) during a 5.6-year follow-up [31]. Notably, in this study, Gal-3 was the only biomarker independently associated with an increased risk of developing HF and death in patients with type 2 diabetes mellitus (HR 2.14 (95% CI: 1.18–3.91)).

In the literature, data are available regarding the prognostic value of Gal-3 as a marker in acute HF [9,32,33]. In our study group, there were only two patients with acute HF. This was most likely due to the fact that we enrolled patients with a first acute MI, in whom successful and complete reperfusion was achieved.

Our current study demonstrated that Gal-3 had a statistically significant diagnostic value in differentiating patients with HF in acute MI. The optimal cut-off value for Gal-3 was determined to be above 10.9 ng/mL, at which a sensitivity of 82.4% and a specificity of 53.6% were achieved. Tymińska et al. revealed that Gal-3 concentration of greater than or equal to 8.74 ng/mL had a sensitivity of 38% and specificity of 81% for prediction of the primary endpoint defined as LVEF below 40% or HF-related hospitalisation or ambulatory diagnosis of HF at one-year follow-up [19]. Gal-3 above 15.97 ng/mL identified HF with preserved EF with 76.0% sensitivity and 71.9% specificity [34].

MI is the main cause of HF [35,36]. After the occlusion of a coronary artery, the ischaemic cardiomyocytes undergo necrosis, and a healing process starts. This process is dynamic, with a sequence of structural and functional changes that includes the removal of necrotic cardiomyocytes to be replaced by a definitive fibrotic scar. Adverse remodelling is characterised by progressive global ventricular dilation and is associated with the temporal evolution of wound healing. While the mechanisms responsible for post-MI ventricular remodelling are multiple, varied, and complex, the magnitude of the inflammatory response during the repair of the infarcted area correlates with the progression of remodelling. Gal-3 plays a multifaceted role in post-infarction wound healing by acting as both a chemotactic factor for macrophage recruitment and a modulator of their polarisation toward the reparative M2 phenotype. This dual action of Gal-3 is pivotal in orchestrating the immune response and tissue repair processes following myocardial infarction, ultimately contributing to cardiac tissue recovery [37].

NT-proBNP is the gold standard biomarker in HF [38,39]. The combined use of Gal-3 and NT-proBNP is important in risk estimation, indicating better efficacy of both markers than each biomarker separately in patients with acute HF [40]. In our study, Gal-3 significantly correlated with NT-proBNP (r = 0.39; *p* < 0.001), which might suggest a shared pathophysiological pathway involving neurohormonal activation and extracellular matrix turnover. A similar relationship was found in the GALAMI study, where log Gal-3 and log NT-proBNP correlated at baseline (r = 0.409; *p* < 0.001), at 1 month (r = 0.514; *p* < 0.001), and at 6 months (r = 0.457; *p* < 0.001) [15]. Gal-3, as a biomarker of fibrosis and remodelling, provides complementary information to NT-proBNP, which reflects volume overload and myocardial stretch. In the review by Srivatsan et al., who evaluated 17 studies on the prognostic value of Gal-3, multivariate analyses showed that inclusion of NT-proBNP in the model attenuated the independent predictive value of Gal-3. However, the combined use of both biomarkers provides a greater prognostic power than either of them alone [41].

No statistically significant correlation was observed in our population between Troponin T concentration and Gal-3 concentration. Gal-3 reflects adverse processes leading to pathological remodelling, such as fibrosis and structural alterations, rather than direct cardiomyocyte necrosis.

We found an association between Gal-3 concentration measured within 7 days from the onset of STEMI/NSTEMI and echocardiographic functional parameters of LV and LA assessed on the same day. In previous studies the association between Gal-3 and imaging biomarkers reflecting LV function post-MI was based on echocardiographic assessment after 6 months of follow-up. Until now, data regarding the early post-acute MI period have been lacking. Andrejic et al. measured Gal-3 concentration on day 1 in central and peripheral arterial blood and on day 30 after MI, in the cubital vein. The baseline Gal-3 concentration was significantly higher in patients who developed LV remodelling after 6 months of follow-up, compared to those who did not, and was inversely associated with the degree of LVEF, while showing a positive association with LA diameter [42]. Similarly, Di Tano and Redondo et al. demonstrated that Gal-3 concentration measured during the acute phase of STEMI was independently associated with adverse LV remodelling at 6 months, suggesting its predictive value for post-infarction HF [15,16]. In studies where cardiac magnetic resonance imaging was used to assess LVEF, patients with elevated baseline plasma Gal-3 concentrations had, on average, significantly lower LVEF (50.3% ± 9.1 vs. 55% ± 8.0; *p* < 0.001) at four months after acute MI [43,44]. Our study confirmed these findings but at an earlier stage of diagnosis and treatment.

Looking at myocardial dysfunction, we went far beyond ejection fraction by using STE, which allowed a more sensitive assessment. We found a Gal-3 concentration-dependent association, whereby higher Gal-3 concentrations were associated with poorer LVEF, GLS, and LARS. GLS is an echocardiographic parameter that assesses myocardial shortening and lengthening along the longitudinal axis during the cardiac cycle. It reflects the function of longitudinal fibres located in the subendocardial layer of the myocardium. This layer is particularly vulnerable to ischaemia, fibrosis, and oxidative stress. Consequently, even subtle myocardial injury leads to a reduction in GLS values before a decline in LVEF becomes apparent. GLS is also correlated with the presence and extent of myocardial fibrosis on cardiac magnetic resonance imaging [45]. LARS is an echocardiographic parameter that assesses LA reservoir function, crucial for diastolic myocardial dysfunction assessment. It is a sensitive marker of early alterations in cardiac haemodynamics and has prognostic significance for HF after STEMI [46].

In this study, a linear regression analysis identified Gal-3 and Troponin T concentrations as independent predictors of echocardiographic indices of myocardial function. The previously cited Tymińska et al. also investigated associations of Gal-3 concentration with echocardiographic parameters of LV systolic and diastolic dysfunction at baseline and at one year. In contrast to our study, there was no clear association between Gal-3 and LVEF, LV and LA dimensions, LV hypertrophy, and e’ and E/e’ [19]. This difference may be explained by the fact that E/e′ ratio and LA size primarily reflect the established abnormalities of LV diastolic function, which often become evident in more advanced stages of myocardial impairment. GLS and LARS are more sensitive echocardiographic indices that can detect subclinical myocardial impairment of the LV and LA, respectively. The ability to capture early functional changes may account for their stronger association with the subsequent development of HF in our cohort.

In our population, both biomarkers, Gal-3 and GLS, were the strongest predictors of HF development in univariate logistic regression analysis and regression analysis with stepwise selection. The combination of an echocardiographic parameter (GLS) and a biomarker of remodelling (Gal-3) demonstrated a good discriminative capacity (AUC = 0.833) for predicting the development of HF after MI. This model yielded an 83% probability of correctly classifying the patients. Although Gal-3 and echocardiographic parameters have been studied separately, evidence concerning the early post-acute MI period and their combined prognostic value remains limited. Our results provide preliminary evidence to address this gap, showing that the integration of Gal-3 with myocardial deformation parameters (GLS, LARS) offers complementary prognostic information and enables a more comprehensive assessment of early post-infarction remodelling processes.

Previous studies have demonstrated that serial measurements of Gal-3 provide additional prognostic information [42,43,44]. However, this approach may be challenging to implement in routine clinical practice. Our observations indicate that a single Gal-3 measurement obtained during the acute phase of MI, when combined with indices of LV and LA function, may allow for an initial prognostic assessment in these patients.

### Study Limitations

This was a single-centre study and the number of patients included was relatively small. Data from the literature suggests that Gal-3 concentration may be influenced by renal disease and non-cardiac fibrosis. In our study population, only six (6%) patients suffered from chronic kidney disease, stages G3a and G3b. Creatinine was included in the univariate and multivariate regression analyses. Serum sampling for Gal-3 measurements was performed within a relatively wide time window (days 3–7 from the onset of acute MI). This was dependent on the timing of complete reperfusion. Nevertheless, the literature data concerning the time of increase and maximum concentration of Gal-3 in the acute phase of MI are not clear.

## 4. Materials and Methods

### 4.1. Study Population and Design

We prospectively screened 661 consecutive patients with chest pain and suspected acute coronary syndrome who were admitted to the Coronary Care Unit of the Cardiology Department, Centre of Postgraduate Medical Education, Grochowski Hospital in Warsaw, between January 2022 and August 2024. The inclusion criteria were: the first STEMI or NSTEMI treated with pPCI with sinus rhythm and without prior HF. The exclusion criteria were: previous MI, HF, atrial fibrillation, severe valvular heart disease (except functional mitral regurgitation), implanted cardiac pacemaker, prior coronary artery bypass grafting, and inadequate echocardiographic image quality. The diagnosis and management of STEMI and NSTEMI were conducted following the prevailing clinical guidelines [22,47,48]. Only patients who fulfilled the diagnostic criteria for NSTEMI and had no documented history of prior MI, post-infarction ECG or echocardiographic changes, or previous revascularisation (PCI/CABG) were classified as having a first NSTEMI [49].

The decision concerning pPCI and the use of coronary stents was left to the discretion of the treating cardiologists. PCI was considered successful when Thrombolysis in Myocardial Infarction (TIMI) grade 3 flow and residual stenosis below 20% were achieved [50]. In the first stage, pPCI of the infarct-related artery was performed, followed by a second-stage PCI of the remaining arteries, if necessary. Further treatment decisions were made by the attending cardiologist, in accordance with current clinical guidelines. Electrocardiograms, clinical examinations, and routine TTE were performed in each patient during hospitalisation. Demographic data, details on comorbidities and current pharmacotherapy, and clinical and angiographic characteristics were prospectively gathered. Routine laboratory parameters, including complete blood count, creatinine, glucose, lipid profile, electrolytes, and biomarkers such as cardiac Troponin T and NT-proBNP, were measured using standard methods at the hospital laboratory. Additionally, according to study protocol, blood samples for Gal-3 and NT-proBNP measurements were collected on the same day as TTE assessed in the study.

The study endpoint was the new-onset HF during the in-hospital phase. We based the diagnosis of new-onset HF on the current guideline recommendations. New-onset HF was defined as the clinical occurrence of new signs and/or symptoms of HF developing after admission for the first MI in patients without a prior history of chronic HF. The diagnosis required the presence of at least one typical symptom and one objective sign of congestion, necessitating specific medical management such as diuretics or oxygen therapy, accompanied by elevated NT-proBNP levels and echocardiographic evidence of LV dysfunction [38,51].

Treatment decisions were made by the attending physicians, following current clinical guidelines [38,39].

The study was approved by the Centre of Postgraduate Medical Education ethics committee (approval number 109/2021). Informed consent was obtained from all subjects involved in the study.

### 4.2. Biomarker Analysis

Serum samples for Gal-3 and NT-proBNP determination were collected between days 3 and 7 after the onset of STEMI/NSTEMI and after complete PCI. Gal-3 concentration was measured using the Alinity Gal-3 assay (Abbott GmbH & Co. KG, Wiesbaden, Germany), which is a two-step chemiluminescent microparticle immunoassay (CMIA), in serum on the Alinity immunoassay analyser. NT-proBNP concentrations were evaluated using the immunoassay for in vitro quantitative determination using the Elecsys proBNP II (Roche Diagnostics GmbH, Mannhein, Germany) in serum on the cobas immunoassay analyser. Cardiac Troponin T concentrations were determined using the Elecsys Troponin T high-sensitivity STAT assay (Roche Diagnostics) on the cobas analyser.

### 4.3. Echocardiography

TTE was performed using the Vivid 9 system (GE Medical System, Horten, Norway, 2010). Echocardiography was performed between days 3 and 7 after onset of STEMI/NSTEMI and after complete PCI. All the images were stored digitally for later analysis. The data were subsequently transferred for off-line analysis using EchoPack Sw Only BT version 110.0.x, GE. The cardiac dimensions were measured in accordance with the current recommendations [52]. To assess systolic LV function, LVEF and GLS were calculated. LARS was assessed as an echocardiographic marker of left atrial (LA) function. LVEF was calculated by the biplane Simpson method [53]. Apical four- and two-chamber and long-axis views were used for quantification of LV GLS by automated function imaging of two-dimensional STE analysis. For each of the three views, the mean longitudinal strain was calculated according to the current standards [54]. LARS was measured by STE. All LARS measurements were analysed according to the recent consensus document of the EACVI/ASE/Industry Task Force to standardise deformation imaging [54]. The other assessed parameters included: LA indexed volume (LAVi), mitral inflow velocities, and early diastolic tissue velocities at the lateral and medial mitral annulus [55]. Right ventricular function was assessed using tricuspid annular plane systolic excursion (TAPSE) [52]. Biomarker results were unavailable to physicians who performed echocardiographic assessments.

### 4.4. Statistical Analysis

Nominal variables were expressed as counts and percentages, while numerical variables were reported as mean and standard deviation or as median and interquartile range, depending on the normality of the distribution assessed using the Shapiro–Wilk test. Numerical variables were compared using the Student’s *t*-test or the Mann–Whitney *U* test (for differences between two groups) and the Kruskal–Wallis test (for differences between three groups), with the DSCF method applied for multiple comparisons, as appropriate. Categorical variables were compared using Pearson’s chi-squared test or Fisher’s exact test.

To reduce the variability of variables in subsequent analyses (correlations and regressions), appropriate transformations were applied. Variables with right-skewed distributions were log-transformed, and variables with left-skewed distributions were squared. The strength of linear associations between numerical variables was assessed using Pearson’s correlation coefficients.

The relationship between biomarkers (Gal-3, NT-proBNP, Troponin T) and LV function (GLS) as well as LA function (LARS) was investigated by means of multivariable linear regression analysis with a backward selection procedure. A ROC curve analysis was used to determine the cut-off point of Gal-3 for the detection of incident HF. The optimal cut-off point was defined on the basis of Youden’s index. Univariable and multivariable binary logistic regression analyses with backward selection were performed to identify independent predictors of HF development. The initial model included factors recognised as potential predictors (age, Troponin T, Gal-3, GLS, LARS, type 2 diabetes mellitus, creatinine, body mass index). Parameters showing strong collinearity, including LVEF and NT-proBNP, were excluded to avoid model overfitting and instability, given the limited sample size. Above all, NT-proBNP could not be included since it was used as a diagnostic criterion for HF in this study. The significance level for a variable to remain in the model (both linear and logistic) was set at *p* < 0.05. Statistical significance was defined as *p* < 0.05.

All statistical analyses were performed using SAS, version 9.4 (SAS Institute Inc., Cary, NC, USA).

## 5. Conclusions

Gal-3 emerged as a valuable biomarker for the early prediction of HF in patients after their first STEMI or NSTEMI, without prior HF. Its concentrations were inversely related to advanced echocardiographic indices of LV and LA function. The combined assessment of Gal-3 and GLS provides a novel, translational biomarker–imaging approach to post-MI prognosis.

## Figures and Tables

**Table 1:** Demographic, laboratory, and clinical characteristics of all patients included in this study and with and without HF.

Variable	All Patients *n* = 105	HF *n* = 34	Without HF *n* = 71	*p* Value
**Demographic parameters**				
Age (years)	61 ± 11.1	63.8 ± 10.6	58.9 ± 11.3	**0.04**
Male gender (%)	81 (77%)	25 (73%)	56 (79%)	0.54
BMI (kg/m^2^)	27 ± 4.0	25.4 ± 3.4	28.6 ± 3.8	**<0.001**
Smoking (%)	46 (43.8%)	16 (47%)	30 (42.2%)	0.93
**Comorbidities**				
DM t.2	22 (21%)	4 (11.8%)	18 (25.3%)	0.11
Arterial hypertension	73 (69.5%)	23 (67.6%)	50 (70.4%)	0.77
CKD	6 (5.7%)	5 (14.7%)	1 (1.4%)	**0.01**
Hyperlipidaemia	79 (75.2%)	28 (82.3%)	51 (71.8%)	0.24
**Laboratory parameters**				
Haemoglobin (g/dL)	14.3 ± 1.4	14.0 ± 1.4	14.5 ± 1.4	0.14
Creatinine (mg/dL), median	0.84 [0.74–0.95]	0.91 [0.81–1.2]	0.81 [0.72–0.93]	**0.005**
Total cholesterol (mg/dL)	200.6 ± 47.4	195 ± 51	203 ± 46	0.38
LDL-C (mg/dL)	122.8 ± 42.6	119 ± 46	125 ± 41	0.52
HDL-C (mg/dL), median	45 [39–55]	48 [41–56]	44 [38–54]	0.19
Triglycerides (mg/dL), median	110 [68–178]	80.5 [62–110]	131 [71–193]	**0.002**
Non-HDL-C (mg/dL), median	88 [67–135]	78 [60–111]	89 [70–144]	0.09
Troponin T * (ng/L), median	1150 [345–3661]	4734 [567–7014]	810 [249–2459]	**<0.001**
NT-proBNP (pg/mL) median	485 [156–1443]	1298 [635–2354]	284 [109–800]	**<0.001**
Galectin-3 (ng/mL) median	11.6 [9.5–13.5]	12.4 [11.5–15.1]	10.8 [8.9–12.6]	**<0.001**
**Acute MI**				
STEMI	59 (56.1%)	24 (70.6%)	35 (49.3%)	**0.04**
NSTEMI	46 (43.9%)	10 (29.4%)	36 (50.7%)	**0.04**
pPCI	105 (100%)	34 (100%)	71 (100%)	
PCI second stage	26 (25%)	8 (24%)	18 (25%)	0.84
Length of all hospital stay (days), median	9 [7–10]	10 [8–13]	9 [7–9]	**<0.001**
CCU (days), median	3 [3–4]	4 [3–5]	3 [3–4]	**<0.001**
**Infarct-related vessel**				
LAD	53 (50.5%)	24 (70.6%)	29 (40.8%)	**0.004**
LCX	27 (25.7%)	6 (17.6%)	21 (29.6%)	0.19
RCA	28 (26.7%)	7 (20,6%)	21 (29.6%)	0.33
**Treatment**				
ACEI	85 (80.9%)	29 (85.3%)	56 (78.9%)	0.43
ARA-II	15 (14.3%)	4 (11.8%)	10 (15.5%)	0.77
β-blockers	88 (83.8%)	29 (85.3%)	59 (83.1%)	0.78
Diuretics	24 (22.9%)	19 (55.9%)	5 (7.0%)	**<0.001**
Aspirin	101 (96.3%)	32 (94.1%)	69 (97.2%)	0.59
Clopidogrel	20 (19.1%)	10 (29.4%)	10 (14.1%)	0.06
Ticagrelor	54 (51.4%)	18 (52.9%)	36 (50.7%)	0.83
Prasugrel	30 (28.6%)	5 (14.7%)	25 (35.2%)	**0.03**
Statins	103 (98.1%)	33 (97.1%)	70 (98.6%)	0.55
SGLT-2 inhibitors	25 (23.8%)	19 (55.9%)	6 (8.4%)	**<0.001**
Aldosterone antagonists	17 (16.2%)	15 (44.1%)	2 (2.8%)	**<0.001**

Data are presented as mean ± SD or numbers (%) or median [IQR]; * maximal value during hospitalisation; Abbreviations: ACEI: angiotensin-converting enzyme inhibitor; ARA-II: angiotensin II receptor antagonist; β-blockers: betablockers; BMI: body mass index; CKD: chronic kidney disease; DM t.2: diabetes mellitus type 2; HDL: high-density lipoprotein cholesterol; LAD: left anterior descending artery; LDL: low-density lipoprotein cholesterol; LCX: left circumflex coronary artery; non-HDLc: non-high-density lipoprotein cholesterol; NSTEMI: non-ST-segment elevation myocardial infarction; NT-proBNP: N-terminal pro B-type natriuretic peptide; pPCI: primary percutaneous coronary intervention; RCA: right coronary artery; SGLT-2: sodium–glucose co-transporter 2; STEMI: ST-segment elevation myocardial infarction.

**Table 2:** Comparison of echocardiographic measurements in patients with and without HF.

Parameter	All Patients *n* = 105	HF *n* = 34	Without HF *n* = 71	*p* Value
LV end diastolic diameter (mm)	45.7 ± 5.4	46.4 ± 5.6	45.4 ± 5.3	0.36
IVS (mm)	12.1 ± 1.7	12.2 ± 1.9	12.1 ± 1.5	0.82
LV posterior wall thickness (mm)	11 ± 1.5	10.7 ± 1.7	11.0 ± 1.4	0.39
LV end diastolic volume (mL)	91.9 ± 25.1	96.5 ± 28.3	89.8 ± 23.3	0.20
LAVi (mL/m^2^), median	31.6 [27.3–40.9]	34.3 [27.3–46.1]	30.6 [27–38]	0.20
LV ejection fraction (%), median	55 [47–60]	43 [38–49]	57 [51–60]	**<0.001**
GLS (%)	−15.5 ± 3.8	−12.9 ± 4.1	−16.8 ± 2.9	**<0.001**
LARS (%)	23.4 ± 6.9	19.8 ± 6.7	25.1 ± 6.4	**<0.001**
RVOT diameter (mm)	29.6 ± 4	28.6 ± 3.8	30.1 ± 4.1	0.07
TAPSE (mm)	20 ± 3.1	19.4 ± 3.5	21.2 ± 2.8	**0.005**
E wave (cm), median	62.7 [50–78]	63.5 [50–78]	60 [50–70]	0.47
A wave (cm), median	70 [56–87]	62.5 [55–80]	70 [60–87]	0.20
E/A, median	0.85 [0.7–1.1]	0.89 [0.7–1.4]	0.82 [0.7–1]	0.13
e’ (cm), median	8 [6–9]	5.5 [5–7]	7 [6–8.2]	**0.002**
E/e’, median	9 [7.8–11]	10 [8.2–12.2]	8.4 [7.4–10]	**0.004**

Data are presented as mean ± SD or median [IQR]; Abbreviations: A, E waves: mitral inflow velocities; e’: mean early diastolic tissue velocity at the lateral and medial mitral annulus; GLS: left ventricle global longitudinal strain; IVS: interventricular septum; LARS: left atrial reservoir strain; LAVi: left atrial indexed volume; LV: left ventricular; RVOT: right ventricular outflow track; TAPSE: tricuspid annular plane systolic excursion.

**Table 3:** Correlation between Gal-3 and NT-proBNP and echocardiographic parameters.

Log Gal-3			Log NT-proBNP	
Variable	r	*p* Value	r	*p* Value
LVEF	−0.27	0.005	−0.41	<0.001
GLS	0.31	0.001	0.38	<0.001
LARS	−0.33	0.001	−0.40	<0.001
Log NT-proBNP	0.39	<0.001		

Abbreviations: Gal-3: galectin-3; GLS: left ventricle global longitudinal strain; LARS: left atrial reservoir strain; LVEF: left ventricular ejection fraction; NT-proBNP: N-terminal pro B-type natriuretic peptide.

**Table 4:** Association between Gal-3 concentrations and LVEF, GLS, and LARS.

	**LVEF < 50%** **Tertile 1**	**50% ≤ LVEF < 60%** **Tertile 2**	**LVEF ≥ 60%** **Tertile 3**	***p* Value**
Gal-3 ng/mL	12.3 [11.1–14.9]	11.2 [9.5–12.8]	10.8 [8.6–12.2]	0.02 1 vs. 3: 0.02
	**GLS > −14.2%** **Tertile 1**	**−14.2% ≥ GLS > −17.7%** **Tertile 2**	**GLS ≤ −17.7%** **Tertile 3**	***p* Value**
Gal-3 ng/mL	12.2 [11.1–14.7]	11.9 [10–14.2]	10.1 [7.9–12]	0.002 1 vs. 3: 0.004
	**LARS ≤ 20.5%** **Tertile 1**	**20,5% < LARS ≤ 26.5%** **Tertile 2**	**LARS > 26.5%** **Tertile 3**	***p* Value**
Gal-3 ng/mL	12.1 [10.3–15.9]	11.9 [9.6–13]	10.1 [8.6–12.1]	0.006 1 vs. 3: 0.006

Abbreviations: Gal-3: galectin-3; GLS: left ventricle global longitudinal strain; LARS: left atrial reservoir strain; LVEF: left ventricular ejection fraction.

**Table 5:** Association between biomarkers and GLS and LARS.

	Regression Coefficient ± SE	Partial Correlation Coefficient	*p* Value
GLS			
Log Troponin T	1.16 ± 0.22	0.47	<0.001
Log Gal-3	2.97 ± 1.09	0.26	0.008
LARS			
Log Troponin T	−1.68 ± 0.42	−0.37	<0.001
Log Gal-3	−6.37 ± 2.08	−0.29	0.003

Abbreviations: Gal-3: galectin-3; GLS: left ventricle global longitudinal strain; LARS: left atrial reservoir strain.

**Table 6:** Risk factors for new-onset HF in acute MI.

Variable	OR [95% CI]	*p* Value
Age	1.041 [1.002–1.082]	0.04
Creatinine	12.7 [1.52–105.6]	0.02
GLS	1.397 [1.204–1.621]	<0.001
LARS	0.880 [0.818–0.946]	<0.001
Troponin T, units = 10	1.004 [1.002–1.006]	<0.001
Gal-3	1.202 [1.056–1.369]	0.005

Abbreviations: Gal-3: galectin-3; GLS: left ventricle global longitudinal strain; LARS: left atrial reservoir.

**Table 7:** Gal-3 and GLS predictors of HF.

Variable	OR [95% CI]	*p* Value	AUC
GLS	1.36 [1.17–1.59]	<0.001	0.833
Gal-3 (log, per 0.1 unit)	1.78 [1.16–2.73]	0.008	

Abbreviations: Gal-3: galectin-3; GLS: left ventricle global longitudinal strain.

## Data Availability

The source data are available in the resources of the Department of Cardiology, Centre of Postgraduate Medical Education, Grochowski Hospital, and in the patients’ documentation.

## Abbreviations

The following abbreviations are used in this manuscript:

MI	Myocardial infarction
HF	Heart failure
Gal-3	Galectin-3
NT-proBNP	N-terminal pro B-type natriuretic peptide
LV	Left ventricular
STEMI	ST-segment elevation myocardial infarction
NSTEMI	Non-ST-segment elevation myocardial infarction
TTE	Transthoracic echocardiography
LVEF	Left ventricular ejection fraction
STE	Speckle tracking echocardiography
SGLT-2	Sodium–glucose co-transporter 2
CKD	Chronic kidney disease
LAD	Left anterior descending artery
GLS	Global longitudinal strain
LARS	Left atrial reservoir strain
TAPSE	Tricuspid annular plane systolic excursion
pPCI	Primary percutaneous coronary intervention
LAVi	Left atrial indexed volume
